# Urinary Human Epididymis Secretory Protein 4 as a Useful Biomarker for Subclinical Acute Rejection Three Months after Kidney Transplantation

**DOI:** 10.3390/ijms20194699

**Published:** 2019-09-22

**Authors:** Soichiro Tajima, Rao Fu, Tomohiro Shigematsu, Hiroshi Noguchi, Keizo Kaku, Akihiro Tsuchimoto, Yasuhiro Okabe, Satohiro Masuda

**Affiliations:** 1 Department of Pharmacy, Kyushu University Hospital, 3-1-1 Maidashi, Higashi-ku, Fukuoka 812-8582, Japan; stajima@pharm.med.kyushu-u.ac.jp (S.T.); shige825@pharm.med.kyushu-u.ac.jp (T.S.); 2 Department of Clinical Pharmacology and Biopharmaceutics, Graduate School of Pharmaceutical Sciences, Kyushu University, 3-1-1 Maidashi, Higashi-ku, Fukuoka 812-8582, Japan; fu.rao.500@s.kyushu-u.ac.jp; 3 Department of Surgery and Oncology, Graduate School of Medical Sciences, Kyushu University, 3-1-1 Maidashi, Higashi-ku, Fukuoka 812-8582, Japan; noguchih@med.kyushu-u.ac.jp (H.N.); k-kaku@med.kyushu-u.ac.jp (K.K.); yokabe1970@gmail.com (Y.O.); 4 Department of Medicine and Clinical Science, Graduate School of Medical Sciences, Kyushu University, 3-1-1 Maidashi, Higashi-ku, Fukuoka 812-8582, Japan; tucimoto@intmed2.med.kyushu-u.ac.jp; 5 Department of Pharmacy, International University of Health and Welfare Narita Hospital, Minami-Aoyama, Minato-ku, Tokyo 107-0062, Japan; 6 Department of Clinical Pharmacy, Faculty of Pharmaceutical Sciences, International University of Health and Welfare Narita Hospital, Minami-Aoyama, Minato-ku, Tokyo 107-0062, Japan

**Keywords:** urinary biomarker, whey-acidic four-disulfide core domain protein 2, acute kidney injury, HE4, WFDC2

## Abstract

Kidney transplantation is the treatment of choice for patients with advanced chronic kidney disease (CKD) and end stage renal disease (ESRD). However, acute rejection (AR) is a common complication in kidney transplantation and is associated with reduced graft survival. Current diagnosis of AR relies mainly on clinical monitoring including serum creatinine, proteinuria, and confirmation by histopathologic assessment in the biopsy specimen of graft kidney. Although an early protocol biopsy is indispensable for depicting the severity of pathologic lesions in subclinical acute rejection (subAR), it is not acceptable in some cases and cannot be performed because of its invasive nature. Therefore, we examined the detection of noninvasive biomarkers that are closely related to the pathology of subAR in protocol biopsies three months after kidney transplantation. In this study, the urinary level of microtubule-associated protein 1 light chain 3 (LC3), monocyte chemotactic protein-1 (MCP-1), liver-type fatty acid-binding protein (L-FABP), neutrophil gelatinase-associated lipocalin (NGAL), and human epididymis secretory protein 4 (HE4) were measured three months after kidney transplantation. Urine samples of 80 patients undergoing kidney transplantation between August 2014 to September 2016, were prospectively collected after three months. SubAR was observed in 11 patients (13.8%) in protocol biopsy. The urinary levels of LC3, MCP-1, NGAL, and HE4 were significantly higher in patients with subAR than in those without, while those of L-FABP did not differ between the two groups. Multivariate regression models, receiver-operating characteristics (ROC), and areas under ROC curves (AUC) were used to identify predicted values of subAR. Urinary HE4 levels were able to better identify subAR (AUC = 0.808) than the other four urinary biomarkers. In conclusion, urinary HE4 is increased in kidney transplant recipients of subAR three months after kidney transplantation, suggesting that HE4 has the potential to be used as a novel clinical biomarker for predicting subAR.

## 1. Introduction

Steady advances in immunosuppression of kidney transplant recipients have resulted in a remarkable decrease in the risk of acute rejection, but long-term outcomes still remain suboptimal [1,2,3]. Subclinical acute rejection (subAR) is defined as histologically proven acute rejection characterized by tubulointerstitial mononuclear infiltration identified from a biopsy specimen in the absence of immediate functional deterioration. Studies of kidney transplant recipients have shown that subAR is associated with an increased risk of graft fibrosis [4,5] and graft loss [6,7,8]. Therefore, early detection and treatment of subAR reduces the incidence of chronic allograft nephropathy and increases graft survival. Also, protocol renal biopsy is needed to monitor patients with stable renal function. However, the invasive nature of biopsy makes it unacceptable in some cases and hence, it cannot be performed serially. Moreover, most protocol biopsies show normal histology, thereby exposing patients to unnecessary biopsy risks. Thus, noninvasive biomarkers are needed to monitor patients who are stable following kidney transplantation.

Various biomarkers for kidney damage have been recently identified such as microtubule-associated protein 1 light chain 3 (LC3), monocyte chemotactic protein-1 (MCP-1), liver-type fatty acid-binding protein (L-FABP), neutrophil gelatinase-associated lipocalin (NGAL), and human epididymis secretory protein 4 (HE4). LC3, which is known as a biomarker for autophagy and has been reported to enhance autophagy and show increased expression during renal injury [9]. MCP-1 is a member of the chemokine family, which plays a role in the recruitment of monocytes to the sites of injury and infection. MCP-1 can also induce the chemotactic effects of monocytes and activate them to promote the production of oxygen-free radicals and the release of lysosomes, which aggravate tissue damage [10]. In addition, MCP-1 is also a urinary biomarker for the early detection of cisplatin-induced nephrotoxicity [11]. L-FABP is a potential novel biomarker for renal dysfunction, expressed in proximal tubules in humans [12]. L-FABP expression attenuates tubulointerstitial damage by reducing oxidative stress [13]. NGAL can easily pass filtration and be conveniently detected in urine in the presence of kidney injury [14]. Urinary NGAL has high sensitivity and specificity for early diagnosis of acute kidney injury (AKI) [14,15,16,17]. HE4, encoded by the whey-acidic four-disulfide core domain protein 2 (WFDC2) gene, is located on chromosome 20q12-12.1 [18]. HE4, a serum biomarker for epithelial ovarian cancer, is an N-glycosylated protein belonging to the whey acidic protein (WAP) family. Recent studies suggest that HE4 could also be a novel biomarker for kidney function [19,20,21,22,23]. In the present study, we examined whether the urinary levels of LC3, MCP-1, L-FABP, NGAL, and HE4 could predict or detect subAR three months after kidney transplantation.

## 2. Results

### 2.1. Patient Characteristics

The characteristics of 80 patients who received kidney transplantation are shown in Table 1, based on the presence or absence of subAR. Eleven patients were diagnosed with subAR by protocol renal biopsy three months after kidney transplantation and 69 patients (no-subAR) were diagnosed with no, or borderline, rejection. The primary diseases observed are listed in Table 1. Age was significantly different between no-subAR and subAR patients. Sex, ABO blood group match, preoperative serum creatinine (Scr) level, preoperative blood urea nitrogen level, and preoperative estimated glomerular filtration rate (eGFR) level did not differ significantly between the no-subAR and subAR patients.

### 2.2. Diagnostic Ability of Urinary Biomarkers

**Figure 1:**
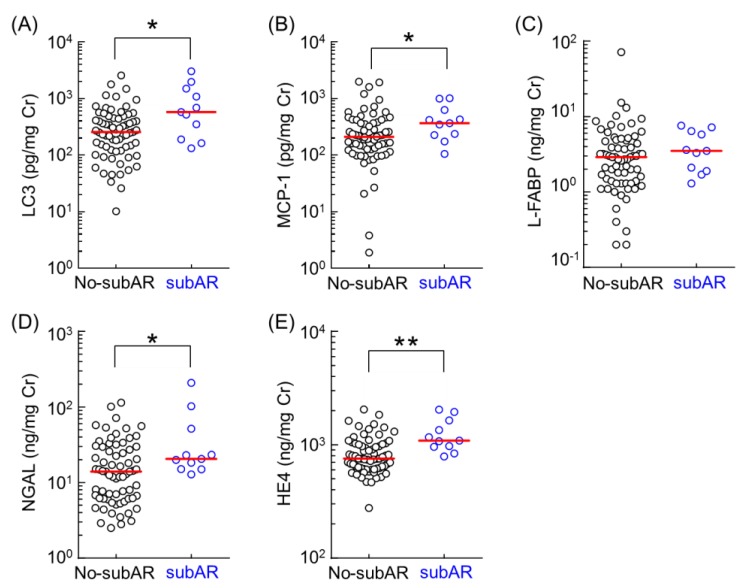
Comparison of the urinary levels of LC3 (**A**), MCP-1 (**B**), L-FABP(**C**), NGAL (**D**), and HE4 (**E**) between the no-subAR group (*n* = 69) and subAR groups (*n* = 11). Data were obtained from urinary samples collected 3 months after kidney transplantation. Bar shows the median value in each group. Data were normalized to urinary creatinine concentration and plotted on a logarithmic Y axis. Statistical analyses were performed using the Mann–Whitney U test. * *p* < 0.05, ** *p* < 0.01.

Five urinary biomarkers were measured in the urine samples, which were collected immediately before the protocol biopsy three months after kidney transplantation. Urinary biomarker levels in no-subAR and subAR patients were summarized (Figure 1). Urinary levels of LC3, MCP-1, NGAL, and HE4 in subAR patients were significantly higher than those in no-subAR patients three months after kidney transplantation. However, urinary levels of L-FABP did not differ between the subAR and no-subAR patients. We performed receiver operating characteristic (ROC) analysis to determine the specificity and sensitivity of urinary biomarkers in the diagnosis of subAR.

The area under the curve (AUC), sensitivity, and specificity obtained from ROC analysis for each urinary biomarker are summarized in Table 2. Based on these results, we focused on the urinary concentrations of HE4 as a useful biomarker to detect subAR three months after kidney transplantation.

**Figure 2:**
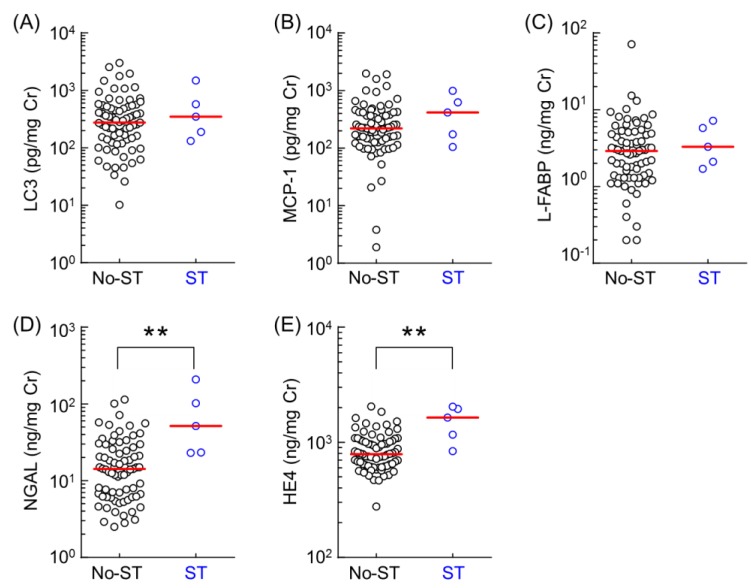
Comparison of the urinary levels of LC3 (**A**), MCP-1 (**B**), L-FABP(**C**), NGAL (**D**), and HE4 (**E**) between the no-ST group (*n* = 75) and ST group (*n* = 5). Data were obtained from urinary samples 3 months after kidney transplantation. Bar shows the median value in each group. Data were normalized against urinary creatinine concentration and plotted on a logarithmic Y axis. Statistical analyses were performed using the Mann–Whitney U test. ** *p* < 0.01.

Since severe tubulitis (ST) in protocol renal biopsy is associated with graft outcome, we examined whether these urinary biomarkers could detect ST three months after kidney transplantation. We compared the urinary level of these biomarkers in five patients with ST (grades t3) and 75 patients with milder grades of tubulitis (grades t0, t1or t2), according to the Banff 2009 classification. The ST group comprised of patients who had developed ST, while the no-ST group comprised of patients who had not developed ST after a three-month postoperative period. Urinary biomarker levels in no-ST and ST patients have been summarized (Figure 2). Urinary levels of NGAL and HE4 in the ST group were significantly higher than those in no-ST patients three months after kidney transplantation. However, urinary levels of LC3, MCP-1, and L-FABP did not differ between the ST and no-ST patients. We performed ROC analysis to determine the specificity and sensitivity of urinary biomarkers in the diagnosis of ST. The AUC, sensitivity, and specificity for ROC curves of each urinary biomarker are summarized in Table 3. The predictive ROC/AUC of urinary HE4 predictive power was 0.875, with sensitivity and specificity at 80.0% and 84.0%, respectively.

## 3. Discussion

In this study, we investigated various candidate urinary biomarkers for the early detection and/or prediction of subAR in patients three months after kidney transplantation. The urinary level of HE4 in our ROC results suggests that it could be a useful biomarker for subAR (AUC = 0.808). However, the urinary level of HE4 was found to be less specific than urinary LC3 and L-FABP in detecting subAR. In this regard, urinary HE4 will be a biomarker that can detect subAR more specifically when used in combination with urinary LC3 or L-FABP. Since tubulitis is regarded as an important histological feature of AR, the urinary biomarkers were also investigated for their ability to detect ST three months after kidney transplantation. Interestingly, HE4 also showed the highest AUC for ST in ROC analysis (AUC = 0.875). Serum HE4 has been used as a clinical diagnostic biomarker for epithelial ovarian cancer and is known to have antiprotease and anti-inflammatory effects [24]. Recently, LeBleu VS et al. [19] identified HE4 as the most upregulated gene in fibrosis-associated myofibroblasts in the kidney, by using α SMA-RFP transgenic mice. HE4 acts as a protease inhibitor by inhibiting the activity of multiple proteases and matrix metalloproteinases, which indirectly inhibit the degradation of type I collagen and results in the occurrence of renal fibrosis [19]. Subsequently, many studies have proven the elevated levels of HE4 to be associated with chronic kidney disease and an advanced stage of renal fibrosis in humans [20,21,22,23]. HE4 was also shown to be negatively correlated with eGFR, with a high diagnostic value of acute and chronic kidney injury with sensitivity and specificity both being 100% [25]. These results suggest that HE4 may be a novel biomarker for early detection of inflammation/fibrosis-related kidney injury.

Previous studies have reported that NGAL is a predictive biomarker of AR [26,27]. Furthermore, Heyne N et al. [28] reported that urinary NGAL can accurately distinguish AR from other causes of AKI in follow-up after kidney transplantation. The present results show that urinary NGAL concentrations are significantly increased in subAR and ST patients compared to no-subAR and no-ST patients. Therefore, it was suggested that urinary NGAL is a biomarker that detects early renal damage as in previous reports. ROC analysis determined the AUC of urinary NGAL for detecting subAR to be 0.715. The only urinary biomarker for which the AUC of the ROC curve for detecting subAR is 0.8 or more and the AUC of the ROC curve for detecting ST is 0.8 or more was HE4. On the other hand, LC3 is an autophagosome membrane protein, which is released during the formation of the autolysosome, making LC3 useful for the evaluation and quantitative comparison of autophagy. Although initially described as a response to starvation, autophagy is now known to be a general cellular response to stress. The correlation between autophagy and kidney function has been recently studied. Autophagy is activated during ischemia–reperfusion (I/R) kidney injury [29] and is induced when renal tubule cells are damaged, showing kidney protection function [9]. In addition, autophagy has also emerged as a key mechanism in orchestrating innate and adaptive immune response to self-antigens [16,30,31,32]. A study has proven that autophagy inducer-like rapamycin can reduce rejection by inducing autophagy to reduce AKI [33]. In the present study, urinary level of LC3 was found to be a useful biomarker for subAR according to ROC analysis (AUC = 0.725). However, the urinary LC3 levels between ST patients and no-ST patients were not significantly different. The non-specificity of urinary LC3 for tubulitis could be because autophagy occurs not only in tubular epithelial cells, but also in various other types of cells. L-FABP has been recognized as a sensitive biomarker of AKI, and its usefulness for predicting patient outcome and for the early diagnosis of AKI has been demonstrated [34,35]. The urinary L-FABP levels between subAR patients and no-subAR patients were not significantly different in this study. In addition, there were also no significant differences in urinary L-FABP levels between ST and no-ST patients. These results were consistent with a clinical study of biomarkers for the early diagnosis of acute rejection after kidney transplantation [27]. In the present study, only 11 patients were diagnosed as subAR based on pathological examination. Furthermore, ST was found in only five patients among 11 subAR patients. Protocol biopsy should be limited in patients with higher risk for AR, due to its invasiveness and risk for bleeding. Noninvasive urinary biomarker HE4, as well as NGAL and LC3, were found to be predictive of subAR. The number of pathological examinations with biopsy specimens might be decreased if these three markers are used to screen for the scale of risk of developing subAR. Despite the limited number of cases used in this study, prescreening with urinary biomarkers to stratify patients based on the need to conduct biopsy may be useful for quality of life of patients by avoiding bleeding risk.

Among the public database of gene expression omnibus (GEO) and the renal gene expression database (RGED), the information for “kidney transplantation” were found in 19 and 28 trials in GEO and RGED, respectively. However, any results corresponding to “subAR” were found in these trials. The HE4 urinary levels of patients diagnosed with subAR by renal biopsy were significantly higher than those of no-subAR patients. Taken together, it is the first report in which urinary HE4 levels at three months after kidney transplantation was suggested to be a predictive biomarker for subAR. Furthermore, of the five urinary biomarkers examined, HE4, NGAL, and LC3 are synthesized in the proximal tubule as well as the distal tubule. On the other hand, MCP-1 and L-FABP are specifically synthesized in the proximal tubule. In this study, it is considered that the acute rejection may be caused by injury in the renal interstitium including distal tubules rather than the proximal tubules. Additionally, acute rejection is considered to be one of the most important factors related to chronic renal graft fibrosis. In a histological examination, Luo et al. [36] reported confirmed association between increased serum HE4 concentrations and renal fibrosis in kidney transplantation, which was further proven by renal biopsy HE4 immunohistochemical (IHC) staining, thereby supporting the present results. In addition, a small amount of urine (approximately 10 μL) may avoid invasive renal biopsy, so the urinary HE4 test is considered cost-effective. Our study has certain limitations. First, because of the small sample size in our study, further prospective analyses with more patients are needed to clarify the significance of our findings. Secondly, our study needs to be verified in an independent cohort because it is the result of a single-center study and the compared groups have age differences. Third, immunosuppressive drugs after kidney transplantation are used for a long time, so it is important to monitor rejection during post-transplant course. Therefore, it is attractive to examine whether urinary HE4 acts as a useful noninvasive biomarker for detecting the late-onset acute rejection several years after kidney transplantation, as well as subAR at the three-month post-transplant protocol examination using biopsy specimens. Analyzing these associations is beneficial to evaluating our findings. In addition, when urinary HE4 levels are used as a biomarker for the diagnosis of kidney injury, the effect of tumors on the results must also be considered. However, in this study, cancer patients were not stratified further and the effect of superposition was not considered. Further studies on larger patient populations and other organ transplant patients are required. In conclusion, the urinary level of HE4 can serve as a sensitive and predictive biomarker for subAR, and urinary HE4-based monitoring of renal functions in kidney recipients may be a convenient and effective way for detecting subAR.

## 4. Materials and Methods

### 4.1. Subjects

A total of 87 adult patients underwent kidney transplantation at Kyushu University Hospital between August 2014 and September 2016. The study excluded 7 patients who developed acute rejection within 3 months of kidney transplantation, those who did not receive protocol renal biopsy, and those with focal segmental glomerulosclerosis (FSGS). Ultimately, our study comprised of 80 kidney transplant recipients. All patients in this study received induction therapy with basiliximab (20 mg/body on days 0 and 4) and a triple-drug regimen comprising of tacrolimus, mycophenolate mofetil, and methylprednisolone. This study was conducted in accordance with the Declaration of Helsinki and its amendments, and was approved by the Institutional Review Board of Kyushu University Graduate School and Faculty of Medicine (approved number: 588-00, 28 July 2014). All patients enrolled in this study gave written informed consent for participation in the study and for the use of their sample.

### 4.2. Urine samples

Spot urine samples were collected before the protocol renal biopsy 3 months after kidney transplantation. All urine samples were stored at −80 °C with protease inhibitor cocktail tablets (Complete Mini, Roche Diagnostics, Mannheim, Germany). Urinary creatinine was determined according to the Jaffe reaction by using the Lab Assay Creatinine kit (Wako Pure Chemical Industries Ltd., Osaka, Japan). The biomarker candidates were measured using commercially available ELISA kits, according to the manufacturer′s instructions. LC3, NGAL, MCP-1, and HE4 were measured using ELISA kits purchased from R & D Systems (Minneapolis, MN). L-FABP level was determined using ELISA kits from CMIC Co., Ltd. (Tokyo, Japan). The level of each urinary biomarker was normalized to urinary creatinine levels to adjust for changes in urine concentration.

### 4.3. Diagnostic Criteria SubAR and Data Collection

A protocol biopsy was performed 3 months after kidney transplantation. All biopsy specimens were scored according to the Banff 2009 classification. There were 69 normal biopsies and 11 with subclinical acute rejection. The diagnostic criteria for subAR were stringent, with the requirement of <10% rise in serum creatinine in the 2 weeks before the protocol biopsy, which was similar to that used by other investigators [37]. Patients with subclinical rejection were classified into those with acute T cell-mediated rejection (TCMR) or acute antibody-mediated rejection (AMR). The clinical information, treatment process, and laboratory data of all patients were obtained from electronic medical records. The preoperative estimated glomerular filtration rate (eGFR) was calculated according to the eGFR equation for the Japanese [38]:

eGFR = 194 × Age − 0.287 × Scr − 1.094 (×0.739, if female)(1)

### 4.4. Statistical Analyses

All statistical analyses were performed using Prism version 8 (GraphPad Software, Inc., San Diego, CA). Mann–Whitney U-test and Kruskal–Wallis tests were used to compare the differences between urinary biomarker levels in patients with/without subAR. We determined receiver operating characteristic (ROC) curves and calculated the area under the curve, 95% confidence intervals (CI), sensitivity, specificity, positive predictive value, negative predictive value, positive likelihood ratio, and negative likelihood ratio. A value of *p* < 0.05 was considered statistically significant.

## Figures and Tables

**Table 1:** Patient characteristics.

Characteristics	No-SubAR (*n* = 69)	subAR (*n* = 11)	*p* Value
Recipient age (years)	42.9 ± 12.7	53.8 ± 14.7	0.019
Recipient sex (male/female)	43/26	5/6	NS
Primary disease (*n*)			NS
Glomerulonephritis	25 (36.2)	5 (45.5)	
Diabetes	17 (24.6)	4 (36.4)	
Polycystic kidney disease	4 (5.8)	2 (18.2)	
Others	23 (33.3)	0 (0.0)	
ABO blood group match (*n*)			NS
Identical	45 (65.2)	6 (54.5)	
Compatible	10 (14.5)	2 (18.2)	
Incompatible	14 (20.3)	3 (27.3)	
Preoperative Scr (mg/dL)	8.5 ± 4.1	8.9 ± 2.9	NS
Preoperative BUN (mg/dL)	59.0 ± 21.2	61.4 ± 20.2	NS
Preoperative eGFR (mL/min/1.73m^2^)	7.6 ± 4.6	5.7 ± 2.7	NS

Data are expressed as mean ± standard deviation, number (%). Abbreviations: Scr, serum creatinine; BUN, blood urea nitrogen; eGFR, estimated glomerular filtration rate.

**Table 2:** Characteristics of the urinary biomarkers in subclinical acute rejection (subAR).

	AUC (95% CI)	Cut-off Value	Sensitivity (95% CI)	Specificity (95% CI)	Positive Predictive Value	Negative Predictive Value	*p* Value
LC3 (pg/mg creatinine)	0.725 (0.554–0.895)	517.9	0.64 (0.31–0.89)	0.78 (0.67–0.87)	0.32	0.93	0.01725
MCP-1(pg/mg creatinine)	0.688 (0.539–0.837)	226.0	0.82 (0.48–0.98)	0.57 (0.44–0.68)	0.23	0.95	0.04655
L-FABP (ng/mg creatinine)	0.606 (0.446–0.767)	7.6	0.09 (0.00–0.41)	0.88 (0.78–0.94)	0.15	1.00	0.2608
NGAL (ng/mg creatinine)	0.715 (0.589–0.842)	12.8	1.00 (0.72–1.00)	0.48 (0.36–0.60)	0.23	1.00	0.0224
HE4 (ng/mg creatinine)	0.808 (0.700–0.916)	789.1	1.00 (0.72–1.00)	0.54 (0.41–0.66)	0.26	1.00	0.001113

**Table 3:** Characteristics of the urinary biomarkers in severe tubulitis (ST).

	AUC (95% CI)	Cut-off Value	Sensitivity (95% CI)	Specificity (95% CI)	Positive Predictive Value	Negative Predictive Value	*p* Value
LC3 (pg/mg creatinine)	0.584 (0.339–0.829)	131.7	1.00 (0.48–1.00)	0.24 (0.15–0.35)	0.08	1.00	0.5313
MCP-1 (pg/mg creatinine)	0.632 (0.365–0.899)	417.9	0.60 (0.15–0.95)	0.76 (0.65–0.85)	0.14	0.97	0.3252
L-FABP (ng/mg creatinine)	0.600 (0.388–0.812)	7.2	0.20 (0.00–0.71)	0.86 (0.76–0.93)	0.07	1.00	0.4561
NGAL (ng/mg creatinine)	0.867 (0.745–0.989)	23.0	1.00 (0.48–1.00)	0.72 (0.60–0.82)	0.19	1.00	0.006299
HE4 (ng/mg creatinine)	0.875 (0.731–1.018)	1165.6	0.80 (0.28–0.99)	0.84 (0.74–0.91)	0.25	0.98	0.005248
